# Implementation of IV Push Antibiotics for Outpatients During a National Fluid Shortage Following Hurricane Maria

**DOI:** 10.1093/ofid/ofac117

**Published:** 2022-03-21

**Authors:** Kruti J Yagnik, L Steven Brown, Hala A Saad, Kristin Alvarez, Norman Mang, Cylaina E Bird, Fred Cerise, Kavita P Bhavan

**Affiliations:** 1 Department of Internal Medicine, University of Texas Southwestern Medical Center, Dallas, Texas, USA; 2 Office of Research Administration, Parkland Health, Dallas, Texas, USA; 3 Center of Innovation and Value at Parkland, Parkland Health, Dallas, Texas, USA; 4 Pharmacy Department, Parkland Health, Dallas, Texas, USA

**Keywords:** cost-savings, fluid shortage, high-value care, Hurricane Maria, IV antibiotics, OPAT

## Abstract

**Background:**

Prior to the introduction of intravenous (IV) drip infusion, most IV drugs were delivered in a syringe bolus push. However, intravenous drip infusions subsequently became the standard of care. Puerto Rico is the largest supplier of IV fluid bags and in the aftermath of Hurricane Maria, there was a nationwide fluid bag shortage. This shortage required stewardship measures to maintain the operation of the self-administered outpatient parenteral antimicrobial therapy (OPAT) program at Parkland Health.

**Methods:**

Parkland pharmacists evaluated all self-administered antimicrobials for viability of administration as an IV syringe bolus push (IVP) instead of an IV-drip infusion. Medications deemed appropriate were transitioned to IVP. The hospital EMR was used to identify patients discharged to the OPAT clinic using all methods of parenteral drug delivery. Data was collected for patient demographics, patient satisfaction, and clinical outcomes. Finally cost of care was calculated for IVP and IV drip administration.

**Results:**

One-hundred and thirteen self-administered IVP and 102 self-administered IV drip treatment courses were identified during the study period. Individuals using IVP had a statistically significant decrease in hospital length of stay. Patient satisfaction was greater with IVP and IVP saved 504 liters of normal saline resulting in a savings of $43,652 over 6 months. The 30-day readmission rate and mortality were similar.

**Conclusion:**

The abrupt IV fluid shortage following a natural disaster led to implementation of a high value care model that improved efficiency, reduced costs, and did not affect safety or efficacy.

Before the introduction of intravenous (IV) drip infusions, which use either an unreconstituted drug attached to an IV fluid bag with 50–100 mL of fluid (in the 1970s) or premixed solution, most intravenously administered drugs were delivered in an IV syringe bolus (IVP) using a concentrated drug solution. With the advent of new agents requiring dilution or slower infusion, IV drip infusion became the standard for all medication delivery. While the new process offered convenience, it was also associated with increased costs, leading to studies comparing clinical outcomes of IVP with IV drip infusion for outpatient parenteral antimicrobial therapy (OPAT) in the home setting [1, 2]. While there was no difference found in frequency of adverse events between methods, the IVP method was found to have several advantages including less training time, lower cost of materials, and reduced waste [1, 2]. Despite these findings, IV drip infusion continued to be the preferred method of drug delivery in both inpatient and outpatient settings due to standardized administration of therapy, convenience for pharmacy, longer medication shelf-life, and the ubiquitous use of infusion pumps.

In September 2017, Hurricane Maria made landfall in Puerto Rico, taking a devastating toll on human life and critical infrastructure. As one of the nation’s main suppliers of IV fluid bags, the response to their shortage was incredibly swift [3, 4]. Regulatory agencies and the manufacturing industry worked to increase the supply, while hospitals and providers adapted measures of conservative use. One of the proposed solutions was to administer intravenous antibiotics via the IVP method rather than using the standard IV drip infusion method. The national shortage of IV fluid bags required effective stewardship at Parkland Health to conserve a scarce resource while meeting the clinical needs of both inpatient and ambulatory settings. Parkland’s unique post–acute care OPAT model allows uninsured patients to transition early from hospital to home to complete a prescribed course of self-administered outpatient parenteral antimicrobial therapy (S-OPAT). We describe the process of adopting a high-value care approach with redesign of care delivery to optimize clinical operations of the OPAT program in a resource-limited setting following Hurricane Maria.

## METHODS

Parkland Health serves a largely uninsured or underinsured patient population residing in Dallas County, Texas [5]. The S-OPAT program was developed in 2009 to provide uninsured patients requiring long-term IV antibiotics for complex infections (eg, osteomyelitis and endocarditis) with the ability to transition earlier from hospital to home to complete a prescribed treatment course. More than 6000 patients to date have been discharged from Parkland hospital to the S-OPAT program to successfully complete care. Patients are taught to self-administer IV antibiotics by gravity (ie, without an infusion pump or device) in the hospital and tested for competency before discharge from the hospital to home. An S-OPAT visit is scheduled for each patient to answer questions and address patient safety concerns. Antibiotics are delivered through a peripherally inserted central catheter (PICC) line requiring ~30 minutes to complete a single infusion by gravity [1].

In response to the national IV fluid shortage, Parkland pharmacists evaluated all self-administered antimicrobials for viability of administration as an IVP and transitioned those that were appropriate [6]. These antibiotics were cefazolin, ceftriaxone, cefepime, and daptomycin. No additional agents were converted. Antimicrobials were selected for IV push administration based on a number of key factors, including published safety and efficacy data, as well as extended syringe stability data of 7 days or longer [6, 7].

The electronic medical record (EMR) was used to identify hospitalized patients discharged to the S-OPAT program before and after the change of method from IV drip (11/2016–06/2017) to prefilled syringes for IVP (11/2017–06/2018). All patients had a bone and joint infection. The EMR was used to monitor patients for antibiotic type, changes in antibiotics, duration of therapy, and adverse events. Additional data were gathered from the EMR and maintained in an internal registry. This included hospital length of stay before S-OPAT visit, days until S-OPAT visit, comparison of predischarge teach-back competency ratio sessions, patient demographics (gender, race, age, language, and payor group), and outcomes such as all-cause readmission rate within 30 days of S-OPAT visit, all-cause readmission rate within 1 year of S-OPAT visit, emergency department (ED) visit within 30 days of S-OPAT visit, ED visit within 1 year of S-OPAT visit, and mortality. Central line–associated bloodstream infection (CLASBI) was the only catheter-related outcome collected. Cost data were obtained from pharmacy suppliers. A subset of nurses and patients who had experience with both IVP and IV drip administration methods were surveyed (Supplementary Data) to assess their satisfaction with the program.

Categorical data were summarized using No. (%) and compared using the chi-square test. Continuous data were summarized with mean ± SD and compared using the *t* test. For median (interquartile range [IQR]), we used the Mann-Whitney *U* test for non–normally distributed data. The level of statistical significance for all tests was <.05. SPSS (version 25) was used to perform statistical analysis.

## RESULTS

One hundred five unique treatment courses were self-administered using the alternative fluid-saving prefilled syringe drug delivery method (IVP) from November 2017 to June 2018, compared with 95 unique treatment courses that were self-administered using the standard IV drip infusion method from November 2016 to June 2017. Gender, ethnicity/race, type of antibiotic, days until OPAT visit, and payor group were similar between both drug delivery methods (Table 1). Patients were primarily male and Hispanic, and the majority were charity or self-pay. There was a significantly (*P* = .02) older demographic in the prefilled syringe IVP group compared with the standard IV drip infusion group, and no central line bloodstream infection within either group (Table 1). Diabetes was a common comorbidity in both groups, with first inpatient hemoglobin A1c averaging 9.7% and 9.6% in the standard IV drip and IV push groups, respectively (Table 1).

**Table 1.** Demographics of S-OPAT Patients Pre/Post IV Push Implementation; Pre: November 2016 to June 2017; Post: November 2017 to June 2018

	Pre (n = 95)	Post (n = 105)	*P* Value^a^
Gender			
Female	20 (21)	23 (22)	.88
Male	75 (79)	82 (78)	
Race/ethnicity			
White Non-Hispanic	15 (16)	18 (17)	.64
Black Non-Hispanic	11 (12)	11 (10)	
Hispanic	68 (72)	72 (69)	
Other	1 (1)	4 (4)	
Language			
English	52 (55)	45 (43)	.22
Spanish	41 (43)	56 (53)	
Other	2 (2)	4 (4)	
Payor group			
Charity/self-pay	93 (98)	101 (96)	.60
Commercial	2 (2)	3 (3)	
Government	0 (0)	1 (1)	
Diabetic	72 (76)	71 (68)	.20
Age, y	47 ± 13	51 ± 12	.01
BMI, kg/m^2^	28.6 (25.3–32.6)	27.4 (24.1–32.9)	.33
First inpatient A1C	9.8 ± 2.7	9.8 ± 2.6	.87
CLABSI	0 (0)	0 (0)	N/A
Days until S-OPAT visit	7 (6–12)	9 (6–13)	.64
Type of antibiotics (check all that apply)			
Cefazolin	5 (5)	14 (13)	.05
Ceftriaxone	41 (43)	51 (49)	.44
Daptomycin	52 (55)	41 (39)	.03
Cefepime	0 (0)	4 (4)	.12

Abbreviations: AIC, Akaike information criterion; BMI, body mass index; CLABSI, central line–associated bloodstream infection; IV, intravenous; S-OPAT, self-administered outpatient parenteral antimicrobial therapy.

No. (%) uses chi-square test or Fisher exact test; mean ± SD uses *t* test; median (interquartile range) uses Mann-Whitney *U* test.

A statistically significant decrease in median hospital length of stay was observed among patients discharged on the IVP method vs standard IV drip infusion for OPAT (12 vs 11 days, respectively; *P* = .04) (Table 2). The average was reduced from 15 days to 12 days between pre and post, but the median was compared because of non-normal distribution. There was no difference in clinical outcomes between both groups, including 30-day readmission rate, 1-year readmission rate, ED visit within 30 days, ED visit within 1 year, and mortality (Table 2).

**Table 2.** Utilization of Hospital Services for S-OPAT Patients Pre/Post IV Push Implementation

	Pre (n = 95)	Post (n = 105)	*P* Value^a^
Hospital length of stay before S-OPAT visit, d	12 (9–17)	11 (8–15)	.03
All-cause readmission rate within 30 d of S-OPAT visit	10 (11)	11 (11)	.99
All-cause readmission rate within 1 y of S-OPAT visit	31 (33)	35 (33)	.92
ED visit within 30 d of S-OPAT visit	21 (22)	25 (24)	.78
ED visit within 1 y of S-OPAT visit	42 (44)	45 (43)	.85
Mortality	4 (4)	7 (6)	.75

Abbreviations: ED, emergency department; IV, intravenous; S-OPAT, self-administered outpatient parenteral antimicrobial therapy.

No. (%) uses chi-square test or Fisher exact test; mean ± SD uses *t* test; median (interquartile range) uses Mann-Whitney *U* test.

The other outcome directly attributable to the route of OPAT administration was the predischarge teach-back competency ratio. While in the hospital, patients were taught how to administer IV antibiotics by both methods by their nurses, and they had to “teach-back” the method to their nurses (before discharge) to demonstrate proficiency in administering their own antibiotics. The number of times they had to teach-back this method was recorded. From a nursing education perspective, the predischarge teach-back competency pass rate was higher with the IVP method (Table 3). This indicates that the IVP method was learned more quickly by patients, and they were able to teach this method back to their nurse in fewer attempts. The IV drip infusion method requires more steps to administer compared with the IVP method. The drip infusion method requires preparation of the drug/compounding, hanging the fluid bag/drug by the gravity method, setting up tubing, attaching the tubing to the PICC line, and then counting the drops of medication so that patients knew how much medication they were getting. The IVP method already has a prefilled syringe of the medication made, so patients only had to take this syringe and inject it into their PICC line. By eliminating multiple steps, the IVP method was significantly easier to learn. There were no differences in antimicrobial usage between the pre and post cohorts. We did not change the way we clinically approached the patients, and there was no change in the infections we treated. The only change that was made was in the way the antibiotics were actually delivered—either by IV drip or IVP.

**Table 3.** Comparison of Predischarge Teach-Back Competency Ratio

	Pre (n = 95)	Post (n = 105)	*P* Value^a^
Teach-back competency ratio			<.01
1:1	5 (5)	10 (10)	
2:1	62 (65)	87 (83)	
3:1	21 (22)	8 (8)	
4:1	5 (5)	0 (0)	
5:1	1 (1)	0 (0)	
6:1	1 (1)	0 (0)	
Teach-back competency ratio group			<.01
≤2:1 (achieves satisfaction in ≤3 attempts)	67 (71)	97 (92)	
≥3:1 (achieves satisfaction in ≥4 attempts)	28 (29)	8 (8)	

No. (%) uses chi-square test.

We also conducted a survey of nurse educators and patients to assess satisfaction with the IVP method and the IV drip infusion method (Supplementary Data). Of the 30 patients eligible to take part in the patient satisfaction survey, 22 (73.3%) completed the interview. When asked which method they would select when requiring IV antibiotic therapy in the future, 96% of participating patients chose the IVP method over the IV drip. Reasons for IVP preference given by patients and nursing staff included reduced administration times (5–10 minutes for push vs 30–60 minutes for slow infusion), convenience, and clear instructions.

The shift to IVP via the S-OPAT program saved 504 liters of normal saline, which, along with a reduction in infusion supplies and direct drug costs, resulted in an additional savings of $43_ _652 over a 6-month period. In addition to conserving IV fluid bags, decreased nursing time required to teach this method to hospitalized patients and reduced length of stay for some patients led to an additional $550_ _000 in cost avoidance over 6 months. The reduced length of stay was due to shorter teaching time for patients to learn the IVP method compared with IV drip.

## DISCUSSION

The abrupt IV fluid shortage following Hurricane Maria challenged clinicians to think differently about standard practices and reflect on lessons learned. This shift in practice to a more efficient care delivery method is generalizable and can be more widely adopted even outside the setting of a fluid shortage. While our patient population is skewed to this cohort, our hospital is a safety net setting, so the applicability of this is likely more generalizable and can be accomplished at other health care institutions. There is potential for even greater savings than those reported in the Parkland S-OPAT population because delivery methods for insured patient populations include even more costly devices such as infusion pumps, which the previously described infusion by gravity method in the safety net setting does not.

A theoretical downside to utilizing IV push administration of beta-lactam antibiotics could be a reduction in a key pharmacodynamic parameter tied to efficacy—the percentage of the dosing interval that drug levels remain above the minimum inhibitory concentration of the target pathogen (T > MIC) [8]. However, in Monte Carlo simulations comparing IV push and standard IV drip infusions, there were no or only minor differences in the T > MIC when comparing 5-minute and 30-minute infusions [9].

What started as a response to a national disaster led to identification and implementation of a high-value care model that was found to be safe, effective, and sustainable, without affecting safety, efficacy, or efficiency. An emergency can provide an opportunity to think differently about nonemergent care. The impact of the hurricane allowed an inefficient practice to be illuminated—the unnecessary use of IV fluid bags for S-OPAT. This serves as a reminder for clinicians to continually examine and question processes in daily practice that may not add value or introduce waste. By revisiting an old health care practice and applying it to a transition of care model for uninsured patients in the safety net setting, a sustainable solution was developed in response to the challenge presented by the IV fluid crisis.

Given cost savings, increased patient satisfaction, and equal clinical outcomes, the IV push model is not only a viable alternative initiated in a time of crisis, but preferable in standard situations; it improves utility and provides high-value care.

## Supplementary Material

ofac117_suppl_Supplementary_Data
