# Supplementary material for: Implementation of IV Push Antibiotics for Outpatients During a National Fluid Shortage Following Hurricane Maria
Source: Open Forum Infect Dis. 2022 Mar 21;9(5):ofac117. doi: 10.1093/ofid/ofac117 (PMC9045948; doi:10.1093/ofid/ofac117)
Supplement: ofac117_suppl_Supplementary_Data [file ofac117_suppl_supplementary_data.docx]

Survey for Patients Who Previously Received OPAT via IV Drip and Now Receiving OPAT IV Push Method

We would like to ask you a few questions about your experience with giving yourself IV antibiotic therapy at home. Your answers will help us make the program better for other patients like you in the future. We are happy to help you complete this survey and it will take about five minutes of your time.

Thinking about your ability to give yourself IV antibiotics at home after completing OPAT training, please rate each of the following as

Demographic Questions:

- In your experience with getting IV antibiotics at home who administered the medication. Caregiver or you?
  - If caregiver say, I will be directing to
- Where did you administer the antibiotics?
- Highest level of education completed:
- Are you currently employed?
  - If so, what do you do?
  - How long did it take you to get back to work after giving yourself IV antibiotics with the hanging bag method?
  - How long did it take you to get back to work after giving yourself IV antibiotics with the Syringe Method?
- Previously on _____ drug ____ times a day, now on ______ drug ______ times a day
- Date of when they had the drip method
- How often are you supposed to give yourself IV antibiotics at home in a day for the last infection
- Was the teaching material (in English or Spanish) helpful? Y/N?
- For those who got material in English, was the video helpful?
  - Which did you prefer, the video or the

IV Drip vs. IV Push Questions:

1. How much do you agree with the statement: After going home from the hospital, I (or caregiver) was able to give myself IV antibiotics without any problems.

IVD:

IVP:

1. Did you need to call the OPAT clinic, nurse hotline number, or go to the hospital for help giving yourself IV antibiotics? (y/ n)

IVD:

IVP:

1. If you answered yes to question #2: who did you contact for help? Where did you go for help? How many times have you had to contact one of these places for help?

IVD:

IVP:

1. On average, how long does it take to complete one IV push antibiotic administration at home? Ask the question for IV drip method next.
2. 0 to 10 minutes
3. 11 to 20 minutes
4. 21 to 30 minutes
5. 31 to 60 minutes
6. Greater than 60 minutes
7. On a scale of 1 to 5 (where 1 is worst, 5 is best) how satisfied are you with your experience with IV push:

IV drip:

1. State the top 3 reasons for satisfaction with IVPush OPAT experience.
2. If you had to do OPAT again or knew someone who had to do OPAT, which method would you choose/recommend? Why?
3. State areas that you think we can improve with the IVPush OPAT program.
